# Sensor-Driven RSSI Prediction via Adaptive Machine Learning and Environmental Sensing

**DOI:** 10.3390/s25165199

**Published:** 2025-08-21

**Authors:** Anya Apavatjrut

**Affiliations:** OASYS Research Group, Computer Engineering, Chiang Mai University, Chiang Mai 50200, Thailand; anya@eng.cmu.ac.th

**Keywords:** machine learning, RSSI, LoRaWAN, radio propagation, sensors, RSSI prediction, RSSI estimation, path loss model

## Abstract

Received Signal Strength Indicator (RSSI) prediction is valuable for network planning and optimization as it helps determine the optimal placements of wireless access points and enables better coverage planning. It is also crucial for efficient handover management between cells or access points, reducing dropped connections and improving service quality. Additionally, RSSI prediction supports indoor positioning systems, power management optimization, and cost-efficient network deployment. Path loss models have historically served as the foundation for RSSI prediction, providing a theoretical framework for estimating signal strength degradation. However, modern machine learning approaches have emerged as a revolutionary solution for network optimization, providing more versatile and data-driven methods to enhance wireless network performance. In this paper, an adaptive machine learning framework integrating environmental sensing parameters such as temperature, relative humidity, barometric pressure, and particulate matter for RSSI prediction is proposed. Performance analysis reveals that RSSI values are influenced by environmental factors through complex, non-linear interactions, thereby challenging the conventional linear assumptions of traditional path loss models. The proposed model demonstrates improved predictive accuracy over the baseline, with relative increases in variance explained of 6.02% and 2.04% compared to the baseline model excluding and including environmental parameters, respectively. Additionally, the root mean squared error is reduced to 1.40 dB. These results demonstrate that cognitive methods incorporating environmental data can substantially enhance RSSI prediction accuracy in wireless communications.

## 1. Introduction

A Received Signal Strength Indicator (RSSI) is a crucial metric that measures the power level of a radio signal received by a wireless device. Expressed in decibels (dB) or decibel-milliwatts (dBm), RSSI quantifies the signal strength at the receiving antenna and serves as an indicator of link quality and reliability.

In recent wireless technologies, the RSSI plays a vital role in optimizing network performance. It enables the identification and mitigation of interference sources, enhancing the overall quality of services [1,2]. By leveraging RSSI data, network operators can anticipate signal strength variations and dynamically adapt transmission power, routing strategies, and sleep cycles. This optimization leads to reduced power consumption and improved network reliability [3,4]. Additionally, RSSI information facilitates intelligent decision-making regarding base station placement, antenna configurations, and power management [1,5]. In scenarios where GPS signals are ineffective, the RSSI serves as a valuable alternative for range-based localization [6,7].

Traditional RSSI estimation at varying distances relies on path loss models to characterize signal attenuation between the transmitter and the receiver. However, these models face several limitations in accurately predicting real-world signal behavior. First, radio signals are not only susceptible to path loss but also to various other factors that affect signal degradation, such as fading and multi-path propagation. Additionally, the path loss model’s performance typically depends on the specific environment where it is deployed. As a result, specialized knowledge of radio propagation is required to identify an appropriate model for each specific location. Both empirical models, which rely on statistically collected data, and linear regression methods have been introduced to optimize path loss parameters.

In contrast to conventional approaches that rely on regression to adjust environment-specific parameters in path loss models, this paper introduces a fully dynamic and adaptive machine learning approach that operates independently of traditional modeling, offering greater flexibility and adaptability. Building on the study by González-Palacio et al. [8], which employed multiple linear regressions to model path loss and establish a predictive baseline, this paper introduces a novel adaptive machine learning framework for RSSI prediction using the same dataset. The results demonstrate that the proposed technique improves RSSI prediction accuracy, and this improvement is further enhanced when environmental factors such as temperature, humidity, barometric pressure, and particulate matter (PM2.5) collected from sensors are included as features. These findings suggest that the relationship between the RSSI and environmental parameters may be more complex than previously assumed. Although individual linear correlations between parameters might be weak, analyzing them collectively through machine learning reveals intricate interactions, highlighting the cognitive nature of path loss modeling. This innovative approach enables more accurate and adaptive RSSI prediction, allowing network operators to optimize their systems based on real-time environmental conditions.

## 2. Literature Review

Traditional path loss models have been widely used for RSSI prediction in wireless communications. These models initially focused on basic parameters such as distance and frequency as the primary sources of signal attenuation [9,10]. Over time, researchers incorporated additional factors into these models, such as fading and multi-path effects from obstacles and environmental conditions, through extra path loss exponents in the log-distance path loss model [10,11,12].

The evolution of path loss modeling has led to increasingly specialized models for various environments and conditions. Researchers have developed specific models for urban, forest, and coastal areas [13], as well as indoor, campus, city, and suburban environments [14]. These models account for various factors including antenna characteristics [15], human presence and movement [16], terrain profiles with diffraction and fading [17], foliage thickness [18], tree height, and electromagnetic wave polarization [19].

Despite numerous attempts to create highly accurate path loss models, a significant limitation remains: these models are typically constrained by location-specific environmental characteristics, as multiple factors can affect signal propagation. Empirical path loss models were later developed to account for various environmental factors using extrapolated measurement data [13,20].

Machine learning has revolutionized wireless communication optimization in recent years, offering powerful new solutions across multiple domains. One particularly promising application is in path loss modeling, where machine learning algorithms were used to perform sophisticated regression analysis to optimize environmental parameters to enhance prediction accuracy. Early implementations successfully integrated machine learning techniques with conventional path loss models, enabling more precise parameter tuning based on specific environmental conditions. This hybrid approach represents an advancement in modeling wireless signal propagation and environmental interactions. There are several previous research studies that employed various techniques such as linear regression, support vector regression, random forests [21], and artificial neural networks [22] to account for environmental data and antenna characteristics [8,19,23,24]. Although regression-based path loss modeling may introduce accuracy improvements, the path loss model still relies on basic mathematical forms such as linear or exponential equations, which may not fully capture the complexity of real-world signal behavior.

More recently, researchers have introduced fully adaptive machine learning approaches for RSSI prediction. For instance, in [25], the authors focused on cellular configuration, leveraging antenna characteristics to predict the reference signal received power (RSRP). This method bypasses traditional path loss modeling, offering greater flexibility by avoiding the constraints of conventional models. Machine learning enables the identification of complex, non-linear relationships among environmental variables, resulting in more accurate and adaptive signal strength predictions.

In [26], Goldoni et al. collected an empirical dataset from LoRaWAN sensor nodes deployed in an agricultural field, which included environmental measurements such as temperature, relative humidity, barometric pressure, and soil characteristics. Their study underscored the potential of these variables for developing more accurate path loss and shadowing models. While the paper presented basic analyses of the relationships among these variables, it did not propose a unified model that integrates them for RSSI prediction.

The importance of incorporating environmental parameters into path loss modeling was further emphasized in [8], where multiple linear regressions were applied to enhance traditional log-distance path loss models. The study demonstrated the benefits of integrating environmental measurements to improve prediction accuracy and introduced two distinct modeling approaches to achieve this. Expanding on this research work, which employed multiple linear regression-based path loss model for estimation, this paper adopts a fundamentally different strategy by leveraging the dynamic and adaptive capabilities of machine learning to construct a purely data-driven framework for RSSI prediction. Instead of explicitly predicting the RSSI by modeling path loss, this paper proposes using trained models to capture complex, non-linear interactions among variables, thereby eliminating the reliance on predefined propagation models. This methodology aligns with the approach introduced by Fauzi et al. [25], where antenna characteristics were incorporated as predictive features within a machine learning framework. In contrast, the present study emphasizes environmental parameters as the primary features. Utilizing the same dataset examined by González-Palacio et al. [8], the selected environmental features include temperature, relative humidity, barometric pressure, and particulate matter (PM2.5).

## 3. Methodology Overview

This section outlines the experimental framework, including both hardware and software components. It provides an overview of the dataset, including the collection procedures and parameter characteristics. The specifications of the baseline model are described, followed by the implemented machine learning algorithms, their configurations, and the evaluation metrics employed in this study.

### 3.1. Experimental Framework

The dataset used in this study is derived from the data provided by González-Palacio et al. [8]. The data is collected from a LoRaWAN network consisting of four LoRa nodes communicating with a single gateway using the LoRa radio frequency protocol. A detailed description of the hardware and dataset is presented in this section.

#### 3.1.1. Hardware Description

**Figure 1:**
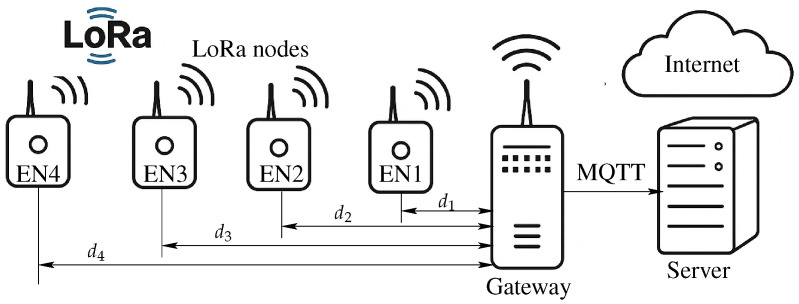
LoRaWAN architecture for dataset retrieval.

According to the hardware description in [8], the four LoRa nodes communicated with a gateway using a radio frequency protocol operating in the 902–928 MHz band. The transmission bandwidth is 125 kHz and cycles through various sub-bands in the ISM US915 band (903.9, 904.1, 904.3, 904.7, 904.9, 905, and 905.1 MHz). The LoRa nodes were positioned at distances of $d_{1}$ = 2.11, $d_{2}$ = 3.42, $d_{3}$ = 5.32, and $d_{4}$ = 8.21 km, respectively, from the gateway, as illustrated in Figure 1.

The LoRa nodes, also referred to as end nodes in the referenced paper, were based on the PyCom LoPy4 platform. These nodes were equipped with an SX1276 (Semtech Corp., Camarillo, CA, USA) radio for LoRa transmission. The antenna used is an omnidirectional PSKN3-900 (Mobile Mark Inc., Itasca, IL, USA) with a peak gain of 3 dBi. The nodes measured environmental variables using various sensors, including a DHT22 (Aosong Electronics Co., Guangzhou, China) for temperature and relative humidity, a BMP280 (Bosch Sensortec GmbH, Reutlingen, Germany) for barometric pressure, an HPMA11500 (Honeywell International Inc., Charlotte, NC, USA) for particulate matter (PM2.5), and an INA219 (Texas Instruments Inc., Dallas, TX, USA) for energy consumption.

The LG308 (Dragino Technology Co., Shenzhen, China) gateway functions as an intermediary between the LoRa nodes and the network server. It is equipped with two radios SX1257 and SX1301 (Semtech Corp., Camarillo, CA, USA) and features an antenna with a peak gain of 4.4 dBi. Upon receiving data from the nodes, the gateway transmits the information to a cloud-hosted message queuing telemetry transport (MQTT) broker, where the payloads are published for subsequent processing.

#### 3.1.2. Dataset Description

The provided dataset contains 930,753 observations recorded in Medellín, Colombia, from October 2021 to March 2022, with a mean sampling interval of 60 s. The data includes the following information, as presented in Table 1 [8].

**Figure 2:**
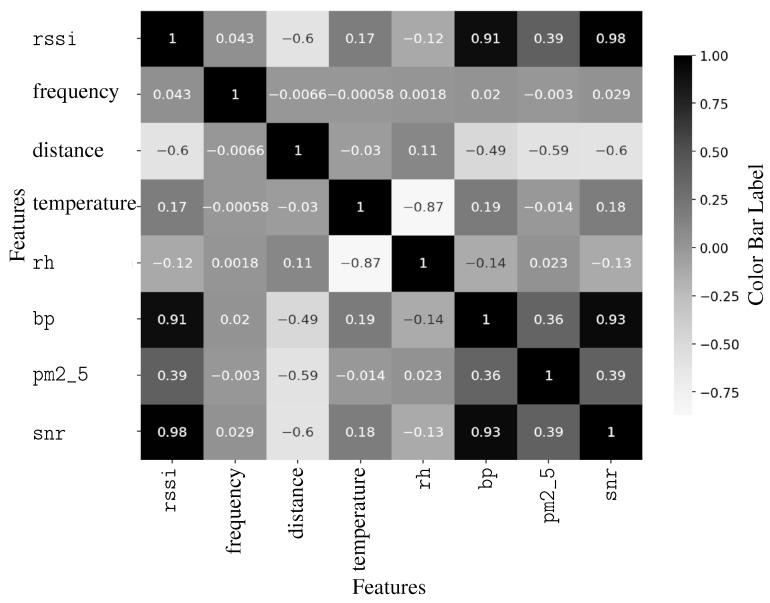
Correlation matrix of variables of interest.

**Figure 3:**
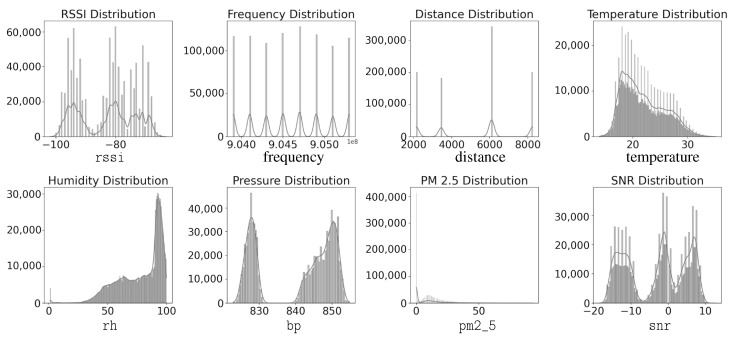
Distribution profiles of variables of interest.

Based on the dataset described in Table 1, eight relevant fields were selected for this study. The selected fields include the target variable for prediction, the Received Signal Strength Indicator (rssi), and key features commonly used in path loss models, such as frequency (*f*) and distance (*d*). Additionally, environmental parameters including temperature (*T*), relative humidity (rh), barometric pressure (bp), and particulate matter (pm2_5) are incorporated to enhance model accuracy. Additionally, the signal-to-noise ratio (snr) is included as part of the training data. Although correlation analysis indicates a very strong relationship between the SNR and the RSSI, suggesting that the SNR alone could serve as an effective predictor of the RSSI, this study includes the SNR as an optional feature. This decision is made to maintain consistency and comparability with the baseline path loss regression model, as described in Equation (3), which already incorporates the SNR as one of its parameters. An initial dataset analysis was conducted in this paper to explore the relationships among these variables, focusing on their correlation and distribution, as illustrated in Figure 2 and Figure 3, respectively.

Figure 2 presents the correlation coefficients between variables. Notably, barometric pressure exhibits a strong positive correlation with the RSSI, with a coefficient of 0.91. This relationship may be influenced by the varying antenna installation heights, as described in the data collection setup. A higher barometric pressure can enhance radio wave propagation through atmospheric ducting effects, which could explain the observed correlation. Additionally, air quality, as represented by the PM2.5 concentration, shows a moderate positive correlation with the RSSI, suggesting that environmental conditions affect signal behavior.

Figure 3 illustrates the distribution profiles of the variables of interest, with the y-axis indicating the frequency of occurrence for each value.The analysis reveals that all variables deviate from normal distributions, with PM2.5 showing the most extreme departure through high positive skewness (1.91) and heavy tails, indicating frequent pollution spikes. Humidity also has some outlier measurements, but they are much fewer. Temperature shows slight positive skewness, indicating occasional hot extremes, while humidity exhibits negative skewness, suggesting more frequent high-humidity conditions.

### 3.2. Baseline Model

Traditionally, the Received Signal Strength Indicator (RSSI) is derived from path loss models, where the log-distance equation serves as the fundamental formulation. To improve the accuracy of predictions, several additional parameters have been introduced, typically involving antenna configurations and environmental factors. Such parameters, as proposed in [8], aim to better align the model with real-world conditions.

The first path loss model mentioned in [8] involves using a multiple linear regression algorithm to determine the coefficients of the well-known *Log-Distance Path Loss Model* (LDPLM), which accounts for path loss along with a random component representing shadowing effects, as shown in Equation (1):

(1) $$\mathrm{LDPLM}=K+10\cdot \gamma \cdot \log_{10}\left(\frac{d}{d_{0}}\right)+\psi .$$

In this equation, *K* represents a scaling factor that adjusts the received signal strength based on system-specific parameters such as antenna gain, system losses, and environmental characteristics; *d* denotes the distance between the transmitter and the receiver; $d_{0}$ is the reference distance, typically set within the far-field region; γ is the path loss exponent, which characterizes the rate at which the signal attenuates with distance; and ψ is a random variable representing shadowing effects, whose statistical distribution is defined as follows in Equation (2):

(2) $$p\left(\psi \right)=\frac{\xi }{\sqrt{2\pi }\cdot \sigma_{\psi }\cdot \psi }\cdot \exp\left(-\frac{{\left(10\cdot \log_{10}\left(\psi \right)-\mu_{\psi }\right)}^{2}}{2\cdot \sigma_{\psi }^{2}}\right),\;\psi >0,$$

where $\xi =\frac{10}{\ln(10)}$, $\sigma_{\psi }$ is the standard deviation of ψ, and $\mu_{\psi }$ is its mean. Based on the results of linear regression analysis, the optimal coefficients that best fit the dataset were specified as γ=2.739 and K=1.75.

The second model proposed in [8] is the *Multiple Linear Regression Model* (MLRM), which extends the LDPLM by incorporating environmental variables such as temperature (*T*), relative humidity (rh), barometric pressure (bp), particulate matter (pm2_5), and the signal-to-noise ratio (snr). These variables are empirically determined through regression analysis to better align the model with real-world signal behavior. The model is expressed as follows:

(3) $$\mathrm{MLRM}={\hat{\beta }}_{0}+10\gamma \log_{10}\left(d\right)+20\log_{10}\left(f\right)+{\hat{\beta }}_{1}T+{\hat{\beta }}_{2}\mathrm{rh}+{\hat{\beta }}_{3}\mathrm{bp}+{\hat{\beta }}_{4}\mathrm{pm}2\_5+{\hat{\beta }}_{5}\mathrm{snr}+\psi .$$

In this equation, ${\hat{\beta }}_{0}$ represents the offset coefficient, which is analogous to *K* in Equation (1). The coefficients ${\hat{\beta }}_{i}$ for i∈{1,…,5} were determined through regression analysis. The model predictors include *f*, the frequency in Hertz (Hz); *T*, the temperature in degrees Celsius; rh, the relative humidity in percentage (%); bp, the barometric pressure in hectopascals (hPa); pm2_5, the concentration of particulate matter with a diameter less than 2.5 μm (μg/m^3^); and snr, the signal-to-noise ratio. The term ψ denotes the shadow fading component, as defined in Equation (2). The coefficient values for each predictor in the MLRM, based on the approach proposed in [25], are summarized in Table 2.

### 3.3. Proposed Model

The model proposed in this paper adopts a novel methodology that utilizes the adaptive capabilities of machine learning to develop a fully data-driven framework for RSSI estimation, referred to as the *Adaptive Machine Learning Model* (AMLM). Rather than relying on traditional path loss equations, the approach employs trained algorithms to capture intricate, non-linear relationships among influencing factors, thereby eliminating the need for predefined signal propagation models.

#### 3.3.1. Model Selection

Low-complexity models, particularly tree-based ensemble methods, were primarily selected in this paper due to the resource constraints of the embedded LoRa gateway framework. This choice is supported by findings reported in [27], indicating that lightweight random forest regressors outperform neural networks with respect to RSSI estimation accuracy, reliability, and computational efficiency. The models explored in this paper range from basic approaches such as linear regression to more advanced regression techniques.

The first basic model selected is *linear regression* (*LR*) [28], which assumes a linear relationship between input features and the target variable, aiming to determine the optimal line that best fits the data. Next, *decision trees* (*DTs*) [29] are included. These models build hierarchical structures by recursively splitting the dataset based on feature values, offering interpretable decision-making capabilities, although they are prone to overfitting.

Tree-based ensemble methods considered such as the *random forest* (RF) [30], *Gradient Boosting* [31], *Extreme Gradient Boosting* (XGBoost or XGB) [32], and *Categorial Boosting* (CatBoost or CB) [33] combine multiple weak learners to form a strong predictive model. The random forest is an ensemble of decision trees that mitigate overfitting and enhance generalization. Gradient Boosting constructs models in a stage-wise manner, iteratively minimizing a loss function to improve performance. XGBoost and CatBoost are advanced implementations of Gradient Boosting, offering additional features and optimizations that improve speed and accuracy. CatBoost was specifically developed to efficiently handle categorical data.

The objective of this study is to evaluate these machine learning algorithms based on their effectiveness in predicting RSSI values. This evaluation aims to assess how well these algorithms can model and capture non-linear relationships among environmental and signal-related variables.

#### 3.3.2. Model Setup

In this study, the framework is implemented using Python libraries, as outlined in Table 3. For ensemble and boosted tree methods, a total of 100 base estimators with a maximum depth of 5 are employed. To ensure reproducibility, the random seed is fixed at 42. The learning rate for both XGBoost and CatBoost is set to 0.1.

The proposed framework was specifically trained to estimate received signal strength at the gateway. To ensure a robust evaluation, the dataset is divided into the training (75%) and testing (25%) subsets.

### 3.4. Evaluation Metrics

To evaluate the performance of the machine learning algorithms, two key metrics were selected: the Percentage of Variance (PoV) and the Root Mean Squared Error (RMSE). In addition, the Mean Absolute Percentage Error (MAPE) is also presented to provide further insight into the prediction accuracy across different models.

#### 3.4.1. Percentage of Variance

*The Percentage of Variance* (PoV) assesses the percentage of total variance in observed data that can be accounted for by a model’s predictions, providing an indication of the model’s capability to capture underlying patterns and relationships in the data. A higher PoV value suggests a better fit and more precise predictions.

The PoV is calculated using the following equation:

(4) $$\mathrm{PoV}=\left(1-\frac{\sum_{i=1}^{n}{\left(y_{i}-\hat{y}i\right)}^{2}}{\sum_{i=1}^{n}{\left(y_{i}-\bar{y}\right)}^{2}}\right)\times 100\%,$$

where *n* represents the number of observations in the dataset, $y_{i}$ is the actual (observed) value of the dependent variable for the *i*-th observation, ${\hat{y}}_{i}$ denotes the predicted value given by the model, and $\bar{y}$ is the mean of the observed values.

#### 3.4.2. Root Mean Squared Error

*The Root Mean Squared Error* (RMSE) measures the average magnitude of the errors between the predicted and actual values. In the context of RSSI prediction, a lower RMSE value indicates that the predicted distances are, on average, closer to the actual distances.

The RMSE is calculated using the following formula:

(5) $$\mathrm{RMSE}=\sqrt{\frac{1}{n}\sum_{i=1}^{n}{\left(y_{i}-{\hat{y}}_{i}\right)}^{2}},$$

where *n* is the number of observations, $y_{i}$ represents the actual (observed) value of the *i*-th observation, and ${\hat{y}}_{i}$ is the predicted value of the *i*-th observation.

#### 3.4.3. Mean Absolute Percentage Error

*The Mean Absolute Percentage Error* (MAPE) quantifies the average magnitude of prediction errors relative to the actual values and is expressed as a percentage. This characteristic makes it particularly effective for comparing prediction performance across different models. The MAPE is calculated as follows:

(6) $$\mathrm{MAPE}=\frac{1}{n}\sum_{i=1}^{n}\left|\frac{y_{i}-{\hat{y}}_{i}}{y_{i}}\right|\times 100\%,$$

where *n* is the total number of observations, $y_{i}$ denotes the actual value of the *i*-th observation, and ${\hat{y}}_{i}$ represents the corresponding predicted value.

## 4. Results and Evaluation

This section presents a performance analysis of the proposed Adaptive Machine Learning Model (AMLM) model. The first subsection evaluates the prediction performance of the framework across various machine learning algorithms, followed by a comparative analysis of their results. Subsequently, the most effective models are selected and benchmarked against predictive results based on the baseline Multiple Linear Regression Model (MLRM) to assess their relative performance.

### 4.1. Performance Analysis of the Proposed Model

The proposed Adaptive Machine Learning Model (AMLM) for RSSI prediction is developed based on several algorithms, including linear regression (LR), decision trees (DTs), random forest (RFs), Gradient Boosting (GB), Extreme Gradient Boosting (XGBoost or XGB), and Categorical Boosting (CatBoost or CB). The models are trained on input features derived from traditional path loss models, specifically distance (*d*) and frequency (*f*), as well as environmental variables that may influence the RSSI, such as temperature (*T*), relative humidity (rh), barometric pressure (bp), and fine particulate matter with a diameter of 2.5 micrometers or less (pm2_5). Additionally, the signal-to-noise ratio (snr) is incorporated to ensure consistency with the baseline model, thereby enabling fair and meaningful comparisons. Model performance is evaluated using multiple metrics, including the Percentage of Variance (PoV), the Root Mean Square Error (RMSE), and execution time, as presented in Table 4, Table 5 and Table 6, respectively.

#### 4.1.1. Percentage of Variance Analysis

The Percentage of Variance (PoV) results in Table 4 demonstrate that model performance improves significantly with the inclusion of additional environmental features. Traditionally, RSSI prediction relies on distance (*d*) and frequency (*f*), which are core components of path loss models. Using distance alone yields PoV values over 93% across most machine learning algorithms, except for linear regression, which performs poorly. In contrast, frequency (*f*) has minimal predictive value, consistently resulting in PoV values below 1%.

Combining distance and frequency offers a slight performance boost, but the most notable improvements occur when environmental parameters such as temperature, relative humidity, barometric pressure, and particulate matter are included. Among these, relative humidity and particulate matter are particularly influential, likely due to their effects on radio wave absorption and scattering. Incorporating all environmental features leads to the highest overall accuracy, emphasizing the value of a comprehensive feature set.

Extreme Gradient Boosting (XGBoost) stands out as the best-performing algorithm thanks to its gradient boosting framework enhanced by optimized tree pruning and regularization. Since the dataset contains only numerical features, Categorical Boosting is not applicable and offer no added benefit. Accordingly, XGBoost results are highlighted in bold in Table 4.

Using only the fundamental variables commonly found in traditional path loss models, such as frequency (*f*) and distance (*d*), XGBoost achieves a prediction accuracy (PoV) of 93.81%. When environmental variables are added (*f*, *d*, temperature (*T*), relative humidity (rh), barometric pressure (bp), and particulate matter (pm2_5)), accuracy improves to 95.69%. Incorporating the full feature set as defined in Equation (3), which includes the signal-to-noise ratio (snr), further boosts the PoV to 97.86%, demonstrating superior model generalization and fit. Two rows in Table 4 are highlighted in gray to indicate key configurations. These configurations will be referenced in the next section for comparison with RSSI predictions generated using the Multiple Linear Regression Model (MLRM) of path loss, which serves as the baseline.

#### 4.1.2. Root Mean Square Error Analysis

The Root Mean Square Error (RMSE) values presented in Table 5 exhibit a trend consistent with the PoV results. Simpler models and limited feature sets result in higher prediction errors, with linear regression performing the worst. As more features are incorporated, the RMSE decreases across all models. XGBoost consistently achieves the lowest RMSE, particularly when the full feature set is utilized. Specifically, it reaches an RMSE of 1.40 when all parameters defined in Equation (3) are included, indicating minimal prediction error. This outcome reinforces XGBoost’s robustness in handling complex, multi-dimensional data.

#### 4.1.3. Execution Time

Execution time (Table 6) reveals a trade-off between accuracy and computational efficiency. While tree-based ensemble methods like the random forest and Gradient Boosting offer high accuracy, they incur significantly higher delays (e.g., over 68 s with full features). In contrast, XGBoost maintains a balance, achieving top performance with relatively low execution time (e.g., 1.90 s with all features). Linear models are the fastest (e.g., 0.02–0.14 s), but their predictive performance is substantially lower. CatBoost also shows moderate execution time (up to 6.14 s) but does not outperform XGBoost in terms of accuracy.

The implementation based on a Python (version 3.11) script was executed on an Intel Core i7-4790S processor (Intel Corporation, Santa Clara, CA, USA) featuring four physical cores with hyper-threading (eight logical cores total), operating at a 3.20 GHz base frequency with turbo boost up to 4.00 GHz. The CPU includes a multi-level cache system with an 8 MiB shared L3 cache and supports an x86_64 architecture with advanced instruction sets including AVX2 for enhanced mathematical computations.

Overall, based on the proposed adaptive machine learning framework, Extreme Gradient Boosting (XGBoost) emerges as the most effective algorithm for RSSI prediction, offering the best trade-off in terms of accuracy (the highest PoV and the lowest RMSE). XGBoost demonstrates the most favorable balance between execution time and model accuracy, making it the most computationally efficient choice among the evaluated algorithms.

The inclusion of environmental and signal-related features significantly enhances model performance, underscoring the importance of comprehensive feature engineering in wireless signal modeling. Using XGBoost, the experimental results demonstrate that incorporating all environmental parameters as input features yields the highest prediction accuracy among all feature selection strategies, achieving a PoV of 97.86% and an RMSE of 1.40.

### 4.2. Evaluation Relative to the Baseline Model

This section evaluates the RSSI prediction results obtained from the proposed Adaptive Machine Learning Model (AMLM), with a particular emphasis on the Extreme Gradient Boosting (XGBoost) algorithm, which achieved the highest RSSI prediction accuracy among all evaluated models. These results are compared with the predictive results using the Multiple Linear Regression Model (MLRM) mentioned in [8].

The MLRM model, based on Equations (2) and (3), enabled path loss prediction with a PoV of 92.50% and an RMSE of 1.84 dB [8]. Unlike path loss prediction, the focus of this paper is on the Received Signal Strength Indicator (RSSI), which can be derived from path loss using a conversion equation also provided by [8]. The equation is as follows:

(7) PL=ptx+gtx+grx−lrx−rssi.

**Figure 4:**
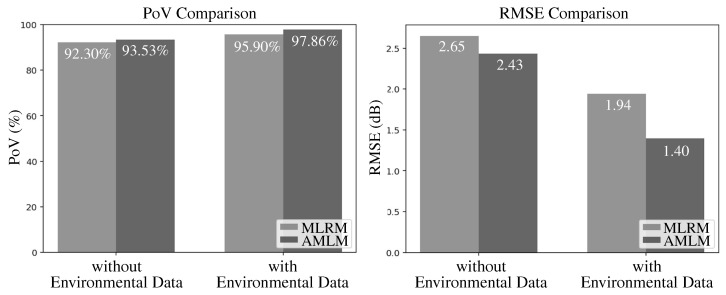
Comparison of the PoV and the RMSE Between the MLRM and the AMLM with and without environmental data for RSSI prediction.

The performance comparison of RSSI prediction between the two models is illustrated in Figure 4. The performance is evaluated under two metrics, namely the Percentage of Variance (PoV) and the Root Mean Square Error (RMSE), and is conducted under two scenarios: one that includes environmental variables and one that excludes them.

Figure 4 indicates that the proposed AMLM demonstrated superior performance, particularly when environmental data is incorporated. Compared to the baseline, the higher PoV achieved by the proposed model indicates its ability to explain a greater percentage of variance in the observed data and to more effectively capture underlying signal patterns. This improvement is consistent across both scenarios regardless of whether environmental factors are considered or not. Regarding the PoV, compared to the prediction when environmental features are excluded (i.e., the MLRM without environmental data, which is equivalent to the LDPLM described in Equation (1)), the PoV increases from 92.30% to 95.90%. When environmental features are included, the PoV further improves from 93.53% to 97.86%, indicating a strong correlation between predicted and actual RSSI values. According to the interpretation guidelines in [34], this level of PoV approaches near-perfect prediction, highlighting the model’s high predictive power. Additionally, the lower RMSE values down to 1.40 dB further validate the enhanced accuracy, indicating that the predicted RSSI values are, on average, closer to the actual measurements.

**Figure 5:**
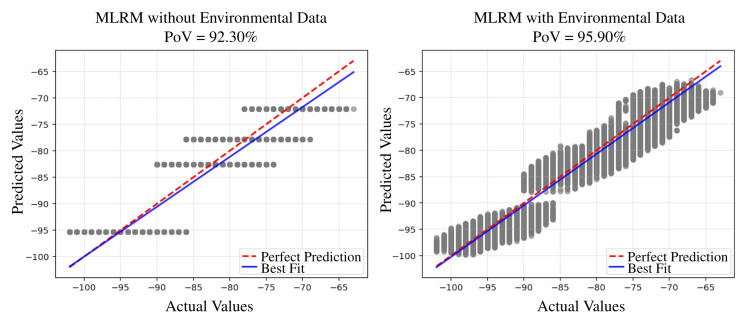
Scatter plot of actual RSSI values vs. predicted RSSI values of the baseline MLRM.

**Figure 6:**
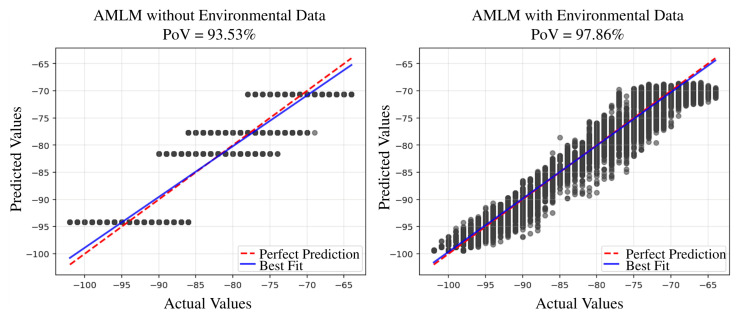
Scatter plot of actual RSSI values vs. predicted RSSI values of the proposed AMLM.

Figure 5 and Figure 6 present scatter plots comparing actual and predicted RSSI values using the Multiple Linear Regression Model (MLRM) and the Advanced Machine Learning Model (AMLM), respectively, under identical conditions. The left plot shows predictions without considering environmental factors, while the right plot incorporates sensing data. In both plots, actual RSSI values are displayed on the x-axis and predicted values on the y-axis. Ideally, perfect predictions would align along the diagonal line, indicating a one-to-one correspondence. Compared to Figure 5, Figure 6 exhibits a tighter clustering around the diagonal line, especially when environmental data is included, highlighting superior predictive accuracy when environmental sensing data is considered.

The integration of environmental factors into the adaptive machine learning model using XGBoost enhances its ability to capture the complex interactions between wireless signal propagation and the surrounding environment. By incorporating variables such as temperature, relative humidity, barometric pressure, and particulate matter (PM2.5), the model can better account for fluctuations in signal strength influenced by these conditions, leading to more accurate RSSI predictions.

**Figure 7:**
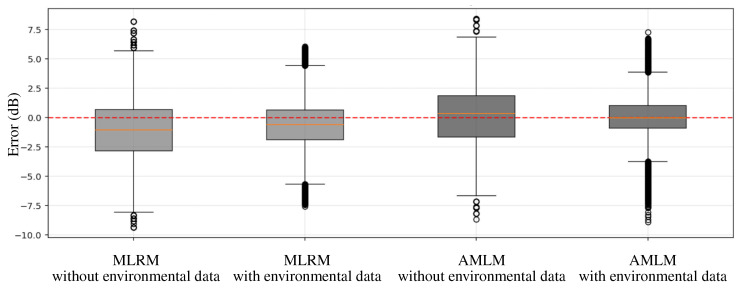
Comparison of prediction error distributions across various models.

Figure 7 compares the distribution of prediction errors across different models. Each box shows how far predicted values deviate from actual values, with the y-axis representing the prediction error. The plot helps assess model accuracy and consistency by highlighting bias, spread, and outliers in prediction performance. The accuracy improvement is evidenced in Figure 7, where the Mean Absolute Percentage Error (MAPE) of the AMLM decreases from 2.42% to 1.37% when environmental factors are included, compared to the baseline model, which shows a reduction from 2.68% to 1.93%. This result demonstrates a meaningful and reliable improvement in performance.

The proposed adaptive machine learning model demonstrates the capability to capture complex, non-linear relationships and interactions among features, characteristics that are often present in real-world wireless signal environments. Unlike traditional path loss modeling, which typically assumes linear relationships and is sensitive to outliers, machine learning approaches such as XGBoost can learn adaptively from data, handle noise more effectively, and automatically model intricate feature interactions. This leads to a better predictive performance, as evidenced by higher PoV and lower RMSE values.

## 5. Conclusions

This paper proposes an innovative approach for predicting the Received Signal Strength Indicator (RSSI) using adaptive machine learning techniques and environmental sensing data. Unlike conventional methods that rely on path loss modeling, which is typically based on linear assumptions, this sensor-driven framework dynamically learns complex, non-linear relationships among environmental variables. These include temperature, relative humidity, barometric pressure, and fine particulate matter (PM2.5), which are collected via a LoRa network.

The adaptive learning capability of the proposed model enables it to capture intricate dependencies that are often overlooked by traditional models. Experimental results demonstrate that the Extreme Gradient Boosting (XGBoost) algorithm achieves strong predictive performance, explaining 97.86% of the variance in the RSSI with a root mean square error of 1.40 dBm. The proposed model demonstrates improved predictive accuracy over the baseline, with relative increases in variance explained of 6.02% (from 92.30% to 97.86%) and 2.04% (from 95.90% to 97.86%) when compared to the baseline model excluding and including environmental parameters, respectively. This outperforms the prediction using a baseline regression-based path loss model. By integrating advanced algorithms with multi-sensor data, the model offers enhanced accuracy and greater adaptability across diverse environmental conditions.

Despite its promising performance, this study’s scope may be constrained by the use of data from only four LoRa nodes, each placed at a distinct distance. Future research can extend this framework by incorporating a wider range of distance measurements and environmental conditions to enhance generalizability. Feature selection may include physical obstacles, atmospheric conditions, electromagnetic interference, and environmental dynamics. Additionally, factors such as antenna characteristics and positioning could further improve the model’s robustness and predictive accuracy.

Overall, this approach introduces a new direction for integrating environmental data into wireless communication systems. Through the use of multiple sensor inputs and sophisticated learning techniques, it offers a deeper understanding of how environmental factors influence signal propagation, with the potential to significantly improve network reliability and overall system performance.

## Figures and Tables

**Table 1:** Dataset details and descriptions.

Column Name	Description
index	Sequential number identifying the corresponding observation.
timestamp	Date and time of the observation in the format of yyyy-mm-dd hh:mm:ss.
device_id	Identifier of the LoRa node defined as EN1, EN2, EN3, or EN4.
distance (*d*)	Distance between the corresponding LoRa node and the gateway in meters.
ht	Antenna height of the transmitter (LoRa node) in meters.
hr	Antenna height of the receiver (gateway) in meters.
ptx	Transmitted radiated power in dBm (fixed at 20 dBm).
ltx	Loss associated with cables and connectors at the transmitter in dB.
gtx	Antenna gain of the transmitter (LoRa node) in dBi.
lrx	Loss associated with cables and connectors at the receiver (gateway) in dB (measured as 4.25 dB).
grx	Antenna gain of the receiver (gateway) measured as 4.161 dBi.
frequency (*f*)	Carrier frequency in Hz, operating in the US 902–928 MHz ISM band.
frame_length	Number of bytes in the current transmission payload.
temperature (*T*)	Temperature in °C.
rh	Relative humidity in %.
bp	Barometric pressure in hPa.
pm2_5	Particulate matter (PM2.5) in μg/m^3^.
rssi	The Received Signal Strength Indicator measured at the gateway in dBm.
snr	The signal-to-noise ratio in dB.
toa	Time on air in seconds.
experimental_pl	Experimental path loss in dB, calculated as ptx + gtx + grx - lrx - rssi.
energy	Energy consumed during the current transmission in Joules.
esp	Effective signal power of the current transmission in dBm.
pn	Noise power in dBm.

**Table 2:** Model predictors and coefficient values for the Multiple Linear Regression Model.

Predictor	Variable	Value	Unit
Intercept	$\hat{K}$	−505.640	dB
Path loss exponent	$\hat{\gamma }$	2.203	-
Temperature	${\hat{\beta }}_{1}$	0.123	dB/°C
Relative humidity	${\hat{\beta }}_{2}$	0.011	dB/%
Barometric pressure	${\hat{\beta }}_{3}$	0.407	dB/hPa
PM2.5	${\hat{\beta }}_{4}$	0.002	dB/μg/m^3^
SNR	${\hat{\beta }}_{5}$	−0.635	dB/dB

**Table 3:** Machine learning libraries.

Technique	Library
LR	sklearn.linear_model.LinearRegression()
DT	sklearn.tree.DecisionTreeRegressor()
RF	sklearn.ensemble.RandomForestRegressor()
GB	sklearn.ensemble.GradientBoostingRegressor()
XGB	xgboost.XGBRegressor()
CB	catboost.CatBoostRegressor()

**Table 4:** PoV-based evaluation predictions across different feature selection methods.

Feature Selection	LR	DT	RF	GB	XGB	CB
*d*	36.48	93.53	93.53	93.53	**93.53**	93.53
*f*	0.19	0.56	0.56	0.56	**0.56**	0.56
*d*, *f*	36.64	93.78	93.78	93.80	**93.81**	93.81
*d*, *f*, *T*	38.92	93.94	93.95	94.06	**94.12**	94.07
*d*, *f*, rh	36.97	94.00	94.01	94.12	**94.19**	94.15
*d*, *f*, bp	86.35	93.82	93.82	93.91	**93.95**	93.93
*d*, *f*, pm2_5	36.79	94.50	94.51	94.58	**94.63**	94.61
*d*, *f*, *T*, rh, bp, pm2_5	86.62	94.80	94.82	95.16	**95.69**	95.38
*d*, *f*, snr, *T*, rh, bp, pm2_5	96.88	97.54	97.58	97.79	**97.86**	97.79

Note: In Table 4, Table 5 and Table 6, two rows are highlighted in gray to indicate key configurations used for comparison in Section 4.2. Additionally, results from the XGBoost model are highlighted in bold to help quickly identify the most relevant findings discussed in the analysis.

**Table 5:** RMSE-based evaluation predictions across different feature selection methods.

Feature Selection	LR	DT	RF	GB	XGB	CB
*d*	7.61	2.43	2.43	2.43	**2.43**	2.43
*f*	9.55	9.53	9.53	9.53	**9.53**	9.53
*d*, *f*	7.61	2.38	2.38	2.38	**2.38**	2.38
*d*, *f*	7.61	2.38	2.38	2.38	**2.38**	2.38
*d*, *f*, *T*	7.47	2.35	2.35	2.33	**2.32**	2.33
*d*, *f*, rh	7.59	2.34	2.34	2.32	**2.30**	2.31
*d*, *f*, bp	3.53	2.38	2.38	2.36	**2.35**	2.35
*d*, *f*, pm2_5	7.60	2.24	2.24	2.22	**2.21**	2.22
*d*, *fT*, rh, bar, pm2_5	3.49	2.18	2.17	2.10	**1.98**	2.05
*d*, *f*, *T*, rh, bp, pm2_5	3.49	2.18	2.17	2.10	**1.98**	2.05
*d*, *f*, snr, *T*, rh, bp, pm2_5	1.69	1.50	1.49	1.42	**1.40**	1.42

**Table 6:** Time delay (*s*) from RSSI estimation with various feature selections.

Feature Selection	LR	DT	RF	GB	XGB	CB
*d*	0.02	0.03	4.72	5.48	**0.87**	3.46
*f*	0.02	0.07	9.58	8.80	**1.23**	3.58
*d*, *f*	0.04	0.15	15.46	16.75	**2.91**	4.61
*d*, *f*, *T*	0.05	0.32	27.18	28.89	**1.67**	4.87
*d*, *f*, rh	0.05	0.39	30.58	31.02	**1.69**	4.84
*d*, *f*, bp	0.05	0.31	26.66	28.38	**1.55**	5.00
*d*, *f*, pm2_5	0.05	0.26	22.86	24.99	**1.64**	5.26
*d*, *f*, *T*, rh, bp, pm2_5	0.11	0.82	58.69	63.21	**2.67**	5.65
*d*, *f*, snr, *T*, rh, bp, pm2_5	0.14	0.96	68.25	74.20	**1.90**	6.14

## Data Availability

The data presented in this study are available on request from the corresponding author.
